# Long-term care need, loneliness, and perceived social isolation during the COVID-19 pandemic: evidence from the German Ageing Survey

**DOI:** 10.1007/s40520-023-02411-0

**Published:** 2023-04-26

**Authors:** André Hajek, Katharina Grupp, Ghazal Aarabi, Razak Mohammed Gyasi, Rosanne Freak-Poli, Benedikt Kretzler, Hans-Helmut König

**Affiliations:** 1 Department of Health Economics and Health Services Research, University Medical Center Hamburg-Eppendorf, Hamburg Center for Health Economics, Martinistr. 52, 20246 Hamburg, Germany; 2 Division of Plastic, Reconstructive and Aesthetic Surgery, University Medical Center Hamburg-Eppendorf, Hamburg, Germany; 3 Department of Periodontics, Preventive and Restorative Dentistry, Center for Dental and Oral Medicine, University Medical Center Hamburg-Eppendorf, Hamburg, Germany; 4 African Population and Health Research Center, Nairobi, Kenya; 5 National Centre for Naturopathic Medicine, Faculty of Health, Southern Cross University, Lismore, NSW Australia; 6 School of Public Health and Preventive Medicine, Monash University, Melbourne, VIC 3004 Australia; 7 School of Clinical Sciences at Monash Health, Monash University, Melbourne, VIC 3004 Australia

**Keywords:** Care degree, Care level, Loneliness, Social isolation, Social exclusion, Functional impairment, Autonomy, Long-term care, Functional dependence

## Abstract

**Background:**

There is a complete lack of studies focusing on the association between care degree (reflecting the long-term care need) and loneliness or social isolation in Germany.

**Aims:**

To investigate the association between care degree and loneliness as well as perceived social isolation during the COVID-19 pandemic.

**Methods:**

We used data from the nationally representative German Ageing Survey, which covers community-dwelling middle-aged and older individuals aged 40 years or over. We used wave 8 of the German Ageing Survey (analytical sample: *n* = 4334 individuals, mean age was 68.9 years, SD: 10.2 years; range 46–100 years). To assess loneliness, the De Jong Gierveld instrument was used. To assess perceived social isolation, the Bude and Lantermann instrument was used. Moreover, the level of care was used as a key independent variable (absence of care degree (0); care degree 1–5).

**Results:**

After adjusting for various covariates, regressions showed that there were no significant differences between individuals without a care degree and individuals with a care degree of 1 or 2 in terms of loneliness and perceived social isolation. In contrast, individuals with a care degree of 3 or 4 had higher loneliness (*β* = 0.23, *p* = 0.034) and higher perceived social isolation scores (*β* = 0.38, *p* < 0.01) compared to individuals without a care degree.

**Discussion/conclusions:**

Care degrees of 3 or 4 are associated with higher levels of both loneliness and perceived social isolation. Longitudinal studies are required to confirm this association.

## Introduction

Due to demographic shifts, a huge increase in the number of individuals in need of care is to be expected in the coming decades in Germany and other high-income countries. Due to the high economic and social burden associated with the need for long-term care for the individuals concerned, their family members, and the insured community, a former study has examined the predictors of the need for care in Germany [1]. More precisely, this former longitudinal study has demonstrated that several factors (such as mobility decline or the occurrence of dementia) can lead to a markedly increased likelihood of the need for care among older adults [1]. Knowledge about the consequences of the need for care (and at each increasing degree) is also of great importance. To date, several international studies have demonstrated an association between impairments in independence (mostly in terms of functional impairment) and loneliness or social isolation [2, 3]. However, there is a complete lack of studies focusing on the association between (explicitly) care degree and loneliness or social isolation in Germany. We assume that having a care degree was particularly challenging in terms of loneliness (subjective discrepancy between real and wished social relationships) and perceived social isolation (an emotion that one does not fully fit into society) in times of the COVID-19 pandemic due to the need of social distancing.

With regard to the course of the pandemic (particularly related to the time of data collection of our current study): national efforts to stop the spread of COVID-19 (e.g., closing schools) began in mid-March 2020. Some restrictions were relaxed in mid-April 2020 and schools reopened in May 2020. Additional restrictions were relaxed in the following months. Since a significant increase in infection rates was observed in the fall of 2020, several restrictions were imposed. Restrictions were relaxed in May 2021.

With regard to the caregiving conditions during the pandemic: prior research [4] demonstrated that informal caregivers of older people with long-term care needs quite often experienced difficulties for themselves in Germany during the pandemic. They also reported that they sometimes felt overwhelmed due to the pandemic [4]. Some caregivers also felt unable to look after their own health [4] and reported feelings of isolation [5]. In addition, former research also showed that caregiving intensity and time increased during the pandemic [6]. Furthermore, mental health strains were often reported by parental caregivers during the pandemic [7]. The pandemic was also perceived as a clear danger for care recipients [6]. Moreover, about one out of five care recipients also had problems in obtaining appropriate care from individuals outside the household during the pandemic [7].

Thus, due to the restricted overall knowledge, our aim was to investigate the association between care degree (reflecting the need for long-term care) and loneliness as well as perceived social isolation during the COVID-19 pandemic. Since loneliness and perceived social isolation can, in turn, lead to premature death [8], knowledge about the association between care degree and these factors is of great importance. Our present study can also serve as a first base for future research in this area.

With regard to our hypotheses and possible explanations: we assume that the need for care (expressed by higher care degrees) is associated with higher levels of both loneliness and perceived social isolation during the times of the COVID-19 pandemic. Possible explanations include the potentially difficult exchange between care recipients and potential private caregivers as well as friends and relatives. This difficult exchange may be explained by the fact that individuals with a high need of care may avoid face-to-face social contacts to avoid infection with COVID-19 (due to the potential severe course of this disease).

With regard to the long-term care system in Germany, some key characteristics are worth describing: a state compulsory insurance system funds costs of care, medication and nursing. This has been introduced in 1995 to assist individuals requiring long-term care. It provides a support (on a medium level) to individuals in need for long-term care—paying particular attention to family care (supplemented by publicly financed services) [9]. Depending on the care degree, a monetary care contribution is computed [10]. This can range up to about EUR 2,000 when an individual has a care degree of 5 (and lives in a nursing home) [10].

## Methods

### Sample

We used data from wave 8 of the German Ageing Survey (DEAS, “Deutscher Alterssurvey”), which included individuals aged 40 years or more who lived in the community (in Germany). The Federal Ministry for Family Affairs, Senior Citizens, Women, and Youth provides funding for the German Aging Survey (BMFSFJ). Fieldwork was conducted by a well-known company (infas). It was conducted between November 2020 and March 2021. A CAPI-per-phone survey was employed, along with a printed drop-off questionnaire for self-completion that asked more sensitive questions such as psychosocial factors. In wave 8, the typical interview lasted roughly 75 min. The DEAS study examines key issues in later life, such as involvement in the work market, well-being, health, or ageism.

The DEAS study has a cohort-sequential design. Since a survey under the COVID-19 pandemic could not be done in a face-to-face interview (as the baseline sample only included address information and not telephone numbers), a new baseline sample could not be added in the most recent wave. Thus, the sample for wave 8 consisted of all individuals who were still accessible and willing to participate in the panel from the baseline samples from 1996 to 2014. The total (gross) sample size was 8379 individuals. A total of 5402 people between the ages of 46 and 100 had valid interviews available (response rate was roughly 65%). In addition, 4419 people (82% of the participants) completed the written drop-off questionnaire. The analytical sample equaled *n* = 4334 individuals as some missing values were present.

The fundamental refusal to participate, and the associated withdrawal of willingness to participate in the panel, were the main causes of non-participation. Younger age, higher education, and good health were all linked to a higher likelihood of participation, while income class, family situation, housing circumstances, and community size were mainly not associated with the likelihood of participation [11]. This indicates that, like many longitudinal studies, the DEAS is affected by the healthy volunteer effect and healthy cohort effect, where healthier (and wealthier) people are more likely to enroll and maintain involvement in research studies. More information regarding the DEAS survey was provided by Klaus et al. [12].

Each participant completed a written informed consent waiver before to the interview. The Helsinki Declaration is followed by the DEAS study. Since the requirements for an ethical approval were not met (e.g., lack of knowledge about the study’s objectives or risk to respondents), an ethical approval was not required for the DEAS study.

### Dependent variables

The De Jong Gierveld loneliness tool was used to measure loneliness [13]. Each of the six items in this tool contains four levels (1 = strongly agree, 2 = agree, 3 = disagree, 4 = strongly disagree). The items were as follows: “I miss the pleasure of the company of others”, “There are plenty of people I can rely on when I have problems”, “I often feel rejected”, “There are many people I can trust completely”, “I miss emotional security and warmth”, and “There are enough people I feel close to”. Thereof, three items were recoded. Thereafter, a mean score was produced (from 1 to 4, with higher scores corresponding to higher loneliness). It is a frequently employed tool with excellent psychometric properties [13]. In our study, Cronbach’s alpha was 0.81 and McDonald’s omega was 0.81.

The second outcome chosen was perceived social isolation. A tool developed by Bude and Lantermann [14] with four items was used for assessment. The items were as follows: “I am worried to be left behind”, “I feel like I do not really belong to society”, “I feel that I am left out” and “I feel excluded from society”. There are four levels (1 = strongly agree, 2 = agree, 3 = disagree, 4 = strongly disagree) to each of the four items. All four items were recoded. The overall score was calculated by averaging the four recoded items. The ultimate score is between 1 and 4, whereby higher values reflect a higher perceived social isolation. Cronbach’s alpha was 0.87 (McDonald’s omega: 0.88) in our present study.

### Independent variables of interest

In the DEAS study, individuals with a care degree were asked: “In which care degree are you currently classified?” [Care degree 1; Care degree 2; Care degree 3; Care degree 4; Care degree 5]. Due to the small number of cases in certain care level degrees (e.g., due to the focus of community-dwelling individuals), we trichotomized the care degree: absence of a care degree (i.e., individuals without a care degree); care degree of 1 or 2; care degree of 3 or higher. However, it should be noted that none of the individuals had a care degree of 5. Thus, the last group solely refers to individuals with a care degree of 3 or 4.

It may be worth clarifying the terms need of care and care degree within the German context. According to § 14 paragraph 1 of the Eleventh Book of the Social Code (“Sozialgesetzbuch”: SGB XI [15]) individuals are in need of care when they have “health-related impairments of independence or abilities and, therefore, require assistance from others. They must be persons who cannot independently compensate for or cope with physical, cognitive or mental impairments or health-related burdens or demands. The need for care must be permanent, probably for at least 6 months, and at least as severe as defined in § 15” (own translation according to the SGB XI [15]). Since 2017, the need for long-term care has been classified into care degrees in Germany (SGB XI, § 15). Following this paragraph, care degrees reflect six life domains:

(i) Mobility (e.g., changing position in bed or climbing stairs),
(ii) Cognitive and communication skills (e.g., recognizing people from the immediate environment, orientation in time and place, understanding requests or taking part in a conversation),
(iii) Behavior and mental health problems (e.g., motor behavioral problems, nocturnal restlessness, self-harming and auto-aggressive behavior),
(iv) Self-care (e.g., using a toilet or washing),
(v) Dealing independently with demands and stresses caused by illness or therapy—and coping with them (e.g., related to medication, wound care or adherence to a diet),
(vi) Shaping everyday life and social contacts (e.g., shaping daily routines and adapting to changes or maintaining contact with people outside the direct environment).

Based on these six life domains, a score was generated which ranges from 0 to 100, whereby higher values reflect a higher degree of dependency. This score was used to classify individuals to a care degree (ranging from 1 to 5):

- Score of 12.5 to < 27: care degree 1 (reflecting minor impairments of independence or abilities)
- Score of 27 to < 47.5: care degree 2 (reflecting considerable impairment of independence or abilities)
- Score of 47.5 to < 70: care degree 3 (reflecting severe impairment of independence or abilities)
- Score of 70 to < 90: care degree 4 (reflecting most severe impairment of independence or abilities)
- Score of 90 to 100: care degree 5 (reflecting most severe impairments of independence or abilities with special requirements for nursing care)

Thus, care degrees reflect the long-term care need.

### Covariates

In agreement with prior literature [16, 17], sociodemographic and health-related covariates were chosen for regression analysis: sex (men; women), age (in years), family situation (single; widowed; divorced; married, living apart from spouse; married, living together with spouse), and education (following the International Standard Classification of Education-97 (ISCED-97) classification [18], which distinguishes between low (0–2), medium (3–4) and high (5–6) education).

Moreover, health-related covariates were selected for regression analysis: self-rated health (from 1 = very good to 5 = very bad; single-item tool), depression (if the score was at least 18 on the 15-item version of the Center for Epidemiologic Studies Depression Scale (CES-D) [19] which ranges from 0 to 45, whereby higher values correspond to more depressive symptoms), and chronic conditions (count score ranging from 0 to 11 covering the following chronic conditions: (i) cardiac and circulatory disorders, (ii) bad circulation, (iii) joint, bone, spinal or back problems, (iv) respiratory problems, asthma, shortness of breath, (v) stomach and intestinal problems, (vi) cancer, (vii) diabetes, (viii) gall bladder, liver or kidney problems, (ix) bladder problems, (x) eye problems, vision impairment, and (xi) ear problems, hearing problems.

### Statistical analysis

Sample characteristics are given stratified by the care degree (absence of a care degree; care degree of 1 or 2; care degree of 3 or 4). We also calculated Cohen’s d for the association between care degree (absence of a care degree; care degree of one or higher) and both outcomes.

In a subsequent step, multiple linear regressions were conducted to examine the association between care degree (also with the trichotomized care degree) and loneliness as well as perceived social isolation. A full-information maximum likelihood (FIML) approach [20] was applied in sensitivity analysis to tackle missing data. A new Stata tool was used to compute McDonald’s omega [21]. The statistical significance was defined as *p* < 0.05 in this study. Stata 16.1 was used to perform statistical analyses (Stata Corp., College Station, Texas).

## Results

### Sample characteristics and bivariate analysis

Sample characteristics (also stratified by the care degree: absence of a care degree; care degree from 1 to 2; care degree of 3 or 4) for the analytical sample are given in Table 1. Mean age equaled 68.9 years (standard deviation, SD: 10.2) in the total sample, ranging from 46 to 98 years, with 51.0% of the individuals being female. There were significant differences in nearly all variables (except for sex) depending on the care degree. For example, among individuals without a care degree, average loneliness level was 1.8 (SD: 0.5) and average perceived social isolation was 1.6 (SD: 0.6). In contrast, among individuals with a care degree from 1 to 2, average loneliness level was 2.0 (SD: 0.6) and average perceived social isolation was 1.9 (SD: 0.7) and among individuals with a care degree of 3 or 4, average loneliness level was 2.2 (SD: 0.5) and average perceived social isolation was 2.3 (SD: 0.8). More details are given in Table 1.

**Table 1:** Sample characteristics among individuals aged 65 years and over (stratified by care degree)

Variables	Absence of a care degree, *n* = 4240 (97.8%)	Care degree from 1 to 2, *n* = 70 (1.6%)	Care degree of 3 or 4, *n* = 24 (0.6%)	*p* value	Total sample *N* = 4334
Loneliness: Mean (SD)	1.8 (0.5)	2.0 (0.6)	2.2 (0.5)	< 0.001	1.8 (0.5)
Perceived social isolation: Mean (SD)	1.6 (0.6)	1.9 (0.7)	2.3 (0.8)	< 0.001	1.6 (0.6)
Sex: *N* (%)				0.76	
Men	2079 (49.0)	32 (45.7)	13 (54.2)		2124 (49.0)
Women	2161 (51.0)	38 (54.3)	11 (45.8)		2210 (51.0)
Age (in years): Mean (SD)	68.6 (10.1)	79.8 (9.0)	78.5 (10.6)	< 0.001	68.9 (10.2)
Education (ISCED-97): *N* (%)				< 0.001	
Low education	155 (3.7)	13 (18.6)	3 (12.5)		171 (3.9)
Medium education	1969 (46.4)	29 (41.4)	10 (41.7)		2008 (46.3)
High education	2116 (49.9)	28 (40.0)	11 (45.8)		2155 (49.7)
Marital status: *N* (%)				< 0.001	
Married, living together with spouse	2974 (70.1)	29 (41.4)	13 (54.2)		3016 (69.6)
Married, living separated from spouse	48 (1.1)	0 (0.0)	0 (0.0)		48 (1.1)
Divorced	388 (9.2)	12 (17.1)	5 (20.8)		405 (9.3)
Widowed	568 (13.4)	26 (37.1)	6 (25.0)		600 (13.8)
Single	262 (6.2)	3 (4.3)	0 (0.0)		265 (6.1)
Self-rated health (from 1 = very good to 5 = very bad): Mean (SD)	2.4 (0.8)	3.6 (0.8)	3.8 (0.9)	< 0.001	2.4 (0.8)
Count score for chronic illnesses: Mean (SD)	2.8 (2.0)	4.9 (2.5)	5.2 (2.5)	< 0.001	2.8 (2.1)
Probable depression (if CES-D ≥ 18): *N* (%)				< 0.001	
Absence of probable depression	4021 (94.8)	57 (81.4)	19 (79.2)		4097 (94.5)
Presence of probable depression	219 (5.2)	13 (18.6)	5 (20.8)		237 (5.5)

One-way ANOVAs or *χ*^2^-tests were conducted, as appropriate (*p* values)

It may be worth noting that the effect size (Cohen’s d) for the differences in loneliness between individuals with and individuals without a care degree was *d* = 0.52 (in absolute terms). Moreover, Cohen’s *d* for the differences in perceived social isolation between individuals with and individuals without a care degree was *d* = 0.71 (in absolute terms).

The average loneliness and isolation levels in the different care degrees were as follows: average loneliness was 2.0 (SD: 0.6) and average perceived social isolation was 1.8 (SD: 0.7) among individuals with care degrees of 1. Moreover, average loneliness was 2.0 (SD: 0.6) and average perceived social isolation was 1.9 (SD: 0.6) among individuals with care degrees of 2. Furthermore, average loneliness was 2.3 (SD: 0.5) and average perceived social isolation was 2.3 (SD: 0.8) among individuals with care degrees of 3. Lastly, average loneliness was 2.0 (SD: 0.7) and average perceived social isolation was 2.2 (SD: 0.9) among individuals with care degrees of 4. Worth repeating: none of the individuals had a care degree of 5.

### Regression analysis

Findings of multiple linear regressions are given in Table 2 (with listwise deletion with FIML to address missings). Loneliness and perceived social isolation served as outcome measures. The trichotomized care degree (absence of a care degree; care degree of 1 or 2; care degree of 3 or 4) served as key independent variable. The models were adjusted for age, sex, marital status, education, self-rated health, number of chronic conditions, and probable depression. *R*^2^ equaled 0.09 and 0.12 with loneliness and perceived social isolation as outcome measures, respectively.

**Table 2:** Care degree, loneliness and social isolation

	Loneliness (with listwise deletion)	Loneliness (with FIML)	Perceived social isolation (with listwise deletion)	Perceived social isolation (with FIML)
Care degree: care degree from 1 to 2 (reference category: absence of a care degree)	0.02 (− 0.12 to 0.17)	0.02 (− 0.12 to 0.16)	0.02 (− 0.13 to 0.17)	0.02 (− 0.13 to 0.17)
Care degree from 3 to higher degrees	0.23* (0.02 to 0.44)	0.23* (0.02 to 0.44)	0.38** (0.12 to 0.65)	0.38** (0.12 to 0.65)
Potential confounders^†^	✔	✔	✔	✔
Individuals	4334	4344	4331	4340
*R*^2^	0.09	0.09	0.12	0.12

Results of multiple linear regressions. Unstandardized beta coefficients are shown. 95% CI are shown in parentheses

***p < 0.001

**p < 0.01

*p < 0.05

^ + ^*p* < 0.10

^†^Potential confounders include age, sex, marital status, education, self-rated health, depression and chronic illnesses

The results of regressions with listwise deletion and with FIML were virtually the same. We, therefore, only report the results with FIML here. Regressions showed that there were no significant differences between individuals without a care degree and individuals with care degree of 1 or 2 in terms of loneliness and perceived social isolation. In contrast, individuals with a care degree of 3 or 4 had higher loneliness (*β* = 0.23, *p* = 0.034) and higher perceived social isolation scores (*β* = 0.38, *p* < 0.01) compared to individuals without a care degree.

## Discussion

Using data from the general adult population aged 40 years and over in Germany, the objective of our study was to examine the association between care degree and loneliness as well as perceived social isolation during the COVID-19 pandemic. Our regressions showed that there were no significant differences between individuals without a care degree and individuals with care degree of 1 or 2 in terms of loneliness and perceived social isolation. In contrast, individuals with a care degree of 3 or 4 had higher loneliness and higher perceived social isolation scores compared to individuals without a care degree. Our current study adds very first insights into the association between care degree and loneliness/isolation in Germany. Since the care degree also reflects, among other things, autonomy, self-care, functional as well as cognitive impairment, our study also contributes to research areas focusing on the link between these factors and loneliness/isolation. In contrast, the existing research is mainly limited to the association between the long-term care need—in terms of functional impairment—and loneliness/isolation (e.g., [2, 3]).

Our initial hypothesis (association between care degree and loneliness/perceived social isolation) was only partly confirmed in regression analysis. The higher loneliness and perceived social isolation scores among individuals with a care degree of 3 or 4 may be explained by the markedly increased difficulty to maintain social relationships due to social distancing during the COVID-19 pandemic. Such individuals (and their relatives and friends) may particularly fear the health consequences of a COVID-19 infection due to their health status. Their social relationships may, therefore, be mainly restricted to professionals (e.g., outpatient care services) during the time of data collection—which may be unsatisfying (in terms of social needs) and may thus intensify feelings of loneliness and social isolation.

It may be worth noting that even if individuals with a care degree of 3 or 4 had already received their first vaccination against the coronavirus, it was presumably the case that their own second vaccination was missing at the time of data collection. This is supported by the fact that only one individual with a care degree of 3 or 4 was interviewed in January 2021 and three individuals with a care degree of 3 or 4 were interviewed in February 2021.

In contrast, we assume that individuals with a care degree of 1 or 2 have not restricted their social activities to such an extent (due to the lower fear of the health consequences of a COVID-19 infection [22] compared to individuals with a care degree of 3 or 4) and may thus did not significantly differ in terms of loneliness and perceived social isolation levels when compared to individuals without a care degree.

As outlined in the introduction: it is worth noting that during the pandemic, informal caregivers struggled with the pandemic [4], and reported feelings of lower health [23] or higher isolation [5]. There is also some evidence showing that caregiving time and intensity increased during the pandemic [6]. In addition, some care recipients struggled with receiving appropriate care from individuals not living in the household during the pandemic [7]. In light of the challenging situation during the pandemic (including social distancing), it appears to be plausible that such challenges for both caregivers and care recipients can also affect relationship quality (e.g., changes in satisfaction as well as an increase in problems with relationship) [24]. This is important because a low relationship quality can contribute to higher loneliness scores in times of the pandemic [25].

We would like to highlight some of our present work’s strengths and shortcomings. A sizable, nationally representative sample served as the source of the data. In addition, our outcome measures were quantified using generally accepted and reliable tools. A FIML approach was used to deal with missing values. It should be acknowledged that the directionality is unclear due to the cross-sectional design used in this study. One cannot rule out the idea that, for instance, high loneliness or isolation levels contribute to increases in care degrees over time. As stated in the Methods section, the response rate in wave 8 was about 65%. This rate is accompanied by a slight sample selection bias in the DEAS study [12]. However, the distribution of sociodemographic (e.g., education, family situation or household size) is very comparable to that of the German population [12].

Furthermore, our study did not include individuals living in institutionalized settings which restrict generalizability to such populations. Individuals living in such settings may also have care degrees of five (and may often suffer from dementia).

## Conclusion and future research

Care degrees of 3 or 4 are associated with higher levels of both loneliness and perceived social isolation (compared to an absence of a care degree). Longitudinal studies are required to confirm these associations. Moreover, if upcoming studies include a sufficient number of individuals, moderating factors (e.g., age group and sex) could be included to explore the potential effect modification of such factors in the associations between care degrees and both loneliness and perceived social isolation in later life. Lastly, studies focusing on individuals living in institutionalized settings would be desirable.

## Data Availability

The data used in this study are third-party data. The anonymized data sets of the DEAS (1996, 2002, 2008, 2011, 2014, 2017, 2020 and 2020/2021) are available for secondary analysis. The data have been made available to scientists at universities and research institutes exclusively for scientific purposes. The use of data is subject to written data protection agreements. Microdata of the German Ageing Survey (DEAS) are available free of charge to scientific researchers for non-profitable purposes. The FDZ-DZA provides access and support to scholars interested in using DEAS for their research. However, for reasons of data protection, signing a data distribution contract is required before data can be obtained. For further information on the data distribution contract, please see https://www.dza.de/en/research/fdz/access-to-data/formular-deas-en-english.
